# Systematic review of AI-based models in pharmacoepidemiology for adverse drug event prediction and detection 

**DOI:** 10.3389/fdsfr.2026.1773186

**Published:** 2026-03-17

**Authors:** Apostolia Karampatea, Konstantinos Kassandros, Theodoros Constantinides, Christos Kontogiorgis

**Affiliations:** Laboratory of Hygiene and Environmental Protection, Medicine Department, Democritus University of Thrace, Alexandroupolis, Greece

**Keywords:** adverse effects, artificial intelligence, drugs reactions, machine learning, polypharmacy

## Abstract

**Introduction:**

Artificial intelligence (AI) has increasingly been applied in pharmacoepidemiology, yet the methodological landscape of adverse drug event (ADE) prediction remains heterogeneous and insufficiently mapped.

**Methods:**

This systematic review aimed to characterize contemporary AI-based approaches used to detect or predict ADEs in real-world clinical data. Following PRISMA 2020 guidelines and a registered protocol (PROSPERO: CRD420251159394), 281 records were screened and 15 studies met the inclusion criteria.

**Results:**

All included studies relied primarily on structured electronic health records (EHRs) or administrative claims, while only a minority incorporated natural language processing (NLP) components, and none used spontaneous reporting systems as the primary analytic datasets. Tree-based ensemble models (e.g., Random Forests, XGBoost) and regularized regression were the most commonly adopted algorithms, whereas deep learning architectures appeared less frequently and typically required temporal or representation-based inputs. Through studies, external or temporal validation was rarely performed and explainability methods were inconsistently applied, limiting generalizability. No standardized benchmarks were identified, and reporting practices varied substantially.

**Discussion:**

Future work should emphasize rigorous validation, transparent model reporting, and the careful integration of NLP and explainability frameworks to support clinically reliable and scalable pharmacoepidemiological applications.

## 1 Introduction

The emergence of artificial intelligence (AI) and machine learning (ML) in biomedical research has increasingly influenced the landscape of pharmacoepidemiology, particularly in the domains of drug safety and pharmacovigilance. Recent studies have reported the growing feasibility of applying ML to detect or predict adverse drug reactions (ADRs) across diverse clinical contexts, including nephrotoxicity (Chiu et al., 2024) bleeding risk associated with SSRIs (Goyal et al., 2023), drug-induced thyroid dysfunction (Lu et al., 2023), liver injury from NSAID interactions (Datta et al., 2021) and myocardial infarction linked to medication exposure (Barbieri et al., 2025). With the increasing availability of real-world data derived from electronic health records (EHRs) and administrative claims, AI-based tools are being used to enhance ADR signal detection and prediction (Li et al., 2014; Segal et al., 2019; Zhao et al., 2015). This methodological shift reflects a broader move from reactive to proactive monitoring in pharmacotherapy, with the aim of improving patient outcomes and supporting regulatory decision-making.

In this review, the term “artificial intelligence” (AI) is used in its broad operational sense, encompassing data-driven computational systems applied to ADE detection and prediction. However, most included studies relied on classical machine-learning (ML) approaches, such as regularized regression and tree-based ensembles, rather than deep learning or fully autonomous AI systems. For conceptual clarity, we therefore distinguish between (i) conventional ML methods (e.g., LASSO, Random Forests, gradient boosting), (ii) deep learning architectures, and (iii) AI-enabled clinical decision support frameworks. This distinction is maintained throughout the manuscript to ensure terminological precision.

Despite the accelerating pace of innovation, a consolidated understanding of how AI models are operationalized within pharmacoepidemiological frameworks remains limited. Current studies vary widely in terms of data sources, methodological rigor, model types, and performance metrics, as reflected in studies using temporal learning approaches (Bagattini et al., 2019), distributed representations of clinical events (Henriksson et al., 2015), natural language processing (NLP)-enhanced literature-based signal discovery (Jeong et al., 2025) and convolution-based risk models for lagged adverse events (Morel et al., 2020). This heterogeneity makes it difficult for clinicians, data scientists, and policymakers to draw systematic conclusions or define best practices. Moreover, the interpretability and external validity of these models key requirements for clinical implementation are often underreported or inconsistently addressed, with only a minority of studies incorporating explicit explainability frameworks (Patterson and Tatonetti, 2024) or external validation across institutions or time periods (Lu et al., 2023; Zhao and Henriksson, 2016).

Given these challenges, a structured synthesis of existing evidence is needed. In this review, adverse drug events (ADEs) are considered an umbrella term that also includes ADRs. This systematic review addresses this gap by mapping the state-of-the-art use of AI models for ADR detection and prediction and by evaluating their clinical relevance, technical transparency, and potential for broader implementation in drug safety monitoring. Specifically, the review aims to identify, categorize, and evaluate the application of AI and ML models in pharmacoepidemiology with a focus on adverse drug effect detection and prediction. It examines how different AI techniques, such as supervised learning algorithms (e.g., LASSO, XGBoost), deep learning architectures, and NLP approaches, have been implemented in real-world datasets (Goyal et al., 2023; Henriksson et al., 2015; Zhao et al., 2015) to support pharmacovigilance activities. The review further explores the types of datasets used, the transparency of the models, the presence or absence of external validation (Lu et al., 2023; Zhao and Henriksson, 2016), and the extent to which these tools have been positioned as decision-support mechanisms within clinical or medication-related decision-making processes (Segal et al., 2019).

## 2 Methods

### 2.1 Study design

This systematic review was conducted in accordance with the Preferred Reporting Items for Systematic Reviews and Meta-Analyses (PRISMA 2020) guidelines. The review protocol was prospectively registered in PROSPERO (ID: CRD420251159394) under the title “Systematic Review of AI-Based Models in Pharmacoepidemiology for Adverse Drug Event Prediction and Detection”. The objective was to identify, classify, and evaluate studies applying artificial intelligence (AI) or machine learning (ML) methods to detect or predict ADE using real-world clinical or pharmacological data.

### 2.2 Search strategy

A comprehensive literature search was performed in PubMed (MEDLINE), covering peer-reviewed articles published between January 2010 and August 2025. The search strategy combined terms related to ADEs and AI approaches, including: “adverse effects”, “pharmacoepidemiology”, “machine learning”, “artificial intelligence”, “LLM”, “LASSO”, “XGBoost”, “SVM”. The search was restricted to English-language publications. Additional records were identified through backward citation screening of included studies and relevant conference proceedings.

### 2.3 Eligibility criteria

Studies were considered eligible according to the criteria of Table 1.

**TABLE 1:** Eligibility criteria.

Inclusion criteria	Exclusion criteria
Applied AI or ML models to detect or predict adverse drug reactions or adverse drug events	Relied exclusively on synthetic, simulated, or fully artificial datasets without real-world data application
Used real-world datasets, including electronic health records (EHRs), administrative claims databases, or pharmacological registries	Did not include an ADE-related outcome
Focused on human patient populations	Did not apply an AI/ML methodology
Used observational or interventional study designs (e.g., cohort, case–control, cross-sectional, or randomized controlled trials)	​

These criteria were applied during the two-stage screening process in the original manuscript. No additional restrictions were applied beyond those criteria.

### 2.4 Study selection

**FIGURE 1:**
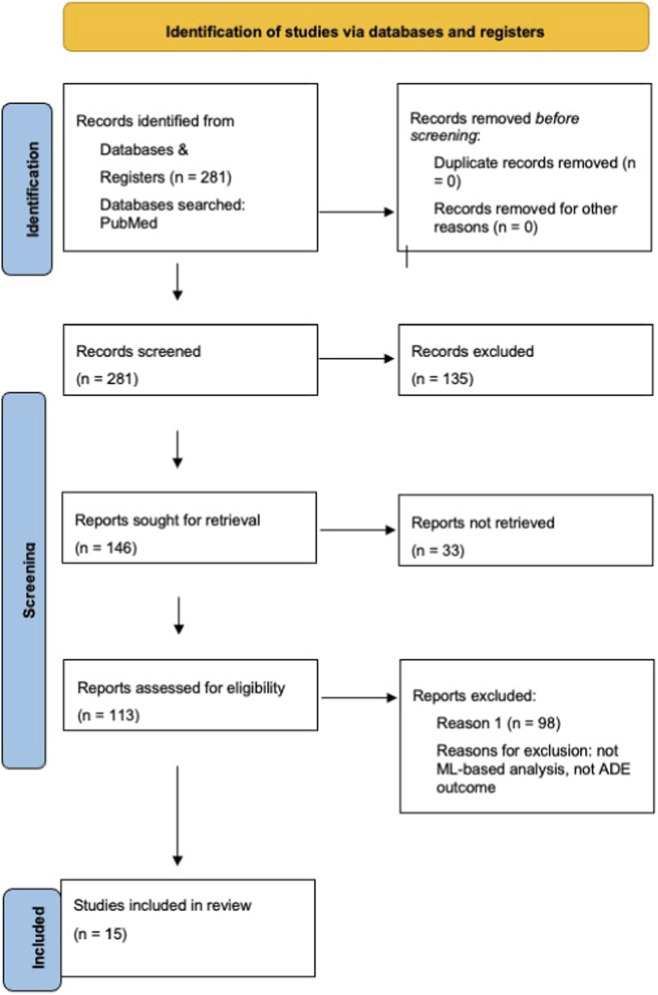
PRISMA 2020 flow diagram summarizing the study selection process.

A total of 281 records were retrieved from database searches. All records underwent title and abstract screening, resulting in 146 articles eligible for further assessment. Full-text review was sought for retrieval (n = 146), of which 33 were not retrievable and 113 were assessed for eligibility. Based on the predefined eligibility criteria, 98 full texts were excluded (including non-ML analysis, absence of ADE outcome, incompatible data sources, insufficient methodological detail). Full-text exclusions were performed strictly according to the predefined eligibility criteria outlined in Section 2.3. As these criteria were applied cumulatively during screening, individual reports could meet more than one exclusion condition; therefore, exclusions are reported in aggregate form in the PRISMA diagram. Ultimately, 15 studies met the inclusion criteria and were included in the qualitative synthesis. The complete study selection process is depicted in the PRISMA 2020 flow diagram (Figure 1).

Two reviewers independently extracted study characteristics (year, clinical domain, population, sample size), dataset type and source (EHR, claims, multi-source designs), AI/ML model families (e.g., logistic regression, ensemble methods, deep learning, NLP components), outcome definitions, performance metrics, validation strategies (internal, temporal, external), key quantitative results, and the primary methodological contribution of each study.

Any discrepancies were resolved through discussion or adjudication by a third reviewer.

Given the anticipated heterogeneity in data sources, modeling techniques, evaluation metrics, and ADE definitions, a narrative synthesis was performed. Findings were thematically stratified by dataset type (EHR/claims only versus EHR plus NLP), methodological focus (model family and feature representation), validation rigor, and clinical domain.

A meta-analysis was not performed due to substantial clinical and methodological heterogeneity across studies.

## 3 Results

### 3.1 Overview of identified studies

Following completion of the systematic search and screening process, a total of 15 studies met the eligibility criteria and were included in the qualitative synthesis. The initial set of retrieved studies exhibited substantial heterogeneity in data sources, analytical objectives, and AI methodologies, as reflected in prior work applying machine learning to diverse ADE contexts such as nephrotoxicity (Chiu et al., 2024), polypharmacy-related events (Dil-Nahlieli et al., 2024), myocardial infarction (Barbieri et al., 2025), liver injury (Datta et al., 2021), thyroid dysfunction (Lu et al., 2023), medication errors (Segal et al., 2019), and ADE detection across multiple clinical settings (Li et al., 2014; Zhao et al., 2015). While the initial intention of the review was to encompass the full breadth of AI applications in adverse drug event (ADE) detection and prediction, the diversity of approaches across the literature necessitated a thematic stratification to enable meaningful synthesis.

A focused analytical framework was therefore adopted, emphasizing studies that used electronic health records (EHRs) or administrative claims data, where predictive models could be evaluated on real-world patient populations and, in some cases, validated across different cohorts or time periods (Lu et al., 2023; Zhao and Henriksson, 2016). This refinement resulted in the identification of 36 potentially relevant studies, of which 21 were excluded after relevance screening based on divergence from this analytical framework. The remaining 15 studies were retained for full synthesis.

### 3.2 Study characteristics and thematic stratification

Across the included studies, two major methodological axes emerged:

1. Models based exclusively on structured EHR or claims data (e.g., Barbieri et al., 2025; Chiu et al., 2024; Goyal et al., 2023; Li et al., 2014; Segal et al., 2019) and
2. Models integrating EHR/claims with NLP-derived representations from clinical text or biomedical literature (e.g., Henriksson et al., 2016; Jeong et al., 2025; Zhao and Henriksson, 2016).

Of the 15 studies, 12 (80%) used only structured EHR or claims, whereas 3 (20%) incorporated NLP components alongside structured data. Importantly, none of the studies employed pharmacovigilance reporting systems (e.g., FAERS, VigiBase) as analytic datasets, indicating a domain-wide shift toward patient-level, real-world clinical data for ADE prediction, a pattern consistent across EHR-based ADE modeling literature (Bagattini et al., 2019; Zhao et al., 2015).

The included studies also covered a broad range of clinical areas, such as nephrotoxicity (Chiu et al., 2024), polypharmacy-related ADEs (Dil-Nahlieli et al., 2024), acute myocardial infarction (Barbieri et al., 2025), thyroid dysfunction (Lu et al., 2023), bleeding risk (Goyal et al., 2023), liver injury and DDI safety (Datta et al., 2021; Jeong et al., 2025), medication error prevention (Segal et al., 2019), and ADE risk modeling in multiple sclerosis (Patterson and Tatonetti, 2024).

### 3.3 Quantitative findings from individual studies

Quantitative extraction across the 15 included studies revealed substantial variability in sample sizes, outcome frequencies, modeling strategies, and performance metrics. Several studies focused on well-defined clinical cohorts and reported detailed incidence rates and discrimination measures. Chiu et al. (2024) examined colistin-induced nephrotoxicity in a cohort of 390 intensive care unit patients, reporting a nephrotoxicity incidence of 13.8%. Among the evaluated models, XGBoost demonstrated the highest predictive performance, achieving an AUROC of approximately 0.81.

Similarly, Dil-Nahlieli et al. (2024) analyzed a large population of 74,553 older adults, of whom 28,412 met criteria for polypharmacy. Within this cohort, 2,023 adverse drug event–related emergency department visits were documented. The evaluated machine-learning–based alert system achieved an AUROC of approximately 0.76 and yielded a net reclassification improvement of around 0.18 when compared with baseline approaches.

Some studies evaluated adverse drug event prediction across multiple datasets and clinical contexts. Zhao et al. (2015) constructed and assessed 27 ADE-specific datasets derived from structured EHR features, consistently demonstrating superior performance of tree-based ensemble models over logistic regression across diverse adverse event types. Comparable patterns were observed in subsequent studies that incorporated more complex feature representations, including Henriksson et al. (2016) and Bagattini et al. (2019).

Henriksson et al. (2016) integrated distributed representations derived from clinical text with structured EHR data, leveraging a corpus comprising approximately 700 million tokens, more than 9,000 ICD-10 codes, and millions of drug records. Their ensemble-based approach using embedding representations improved ADE prediction performance relative to classical baselines. Bagattini et al. (2019) focused on sparse temporal abstractions derived from EHR time-series data, evaluating their methods across 15 ADE datasets, each consisting of several hundred episodes. The proposed temporal transformations consistently enhanced model discrimination compared with non-temporal feature representations.

A related emphasis on controlling clinical complexity was present in Li et al. (2014), who analyzed 316,178 statin users and observed 10,745 myocardial infarctions over more than 1.3 million person-years. Their high-dimensional propensity score–based adjustment strategies strengthened the validity of ADE-related effect estimates by mitigating complex confounding patterns.

Methodological innovation was also evident in studies combining simulation-based experiments with real-world data. Datta et al. (2021) evaluated drug-induced liver injury interactions using simulated cohorts of 4,000 subjects per scenario alongside French national claims data, illustrating how mechanistic signals can complement observational evidence. Morel et al. (2020) similarly employed simulations involving 4,000 individuals to evaluate the ConvSCCS framework before applying it to French SNIIRAM claims data, where convolution-based modeling improved the detection of lagged adverse event risk patterns.

Other investigations emphasized clinical decision support and interpretability. Segal et al. (2019) evaluated a medication-safety clinical decision support system that generated alerts for 0.4% of prescriptions, with 85% of alerts rated as clinically valid and 43% resulting in prescription modification. Patterson and Tatonetti (2024) introduced the KG-LIME framework, demonstrating that explanation complexity could be reduced while preserving fidelity to model predictions.

It is of high interest that in some cases, clinical domains were associated with elevated adverse event risk. Lu et al. (2023) analyzed thyroid dysfunction in 2,544 patients treated with immunotherapies, reporting that 42.5% experienced thyroid-related adverse events and that the best-performing model achieved an AUROC of approximately 0.86. Goyal et al. (2023) evaluated bleeding risk among 11,408 SSRI users, identifying 706 confirmed bleeding events and reporting a top C-statistic of approximately 0.78.

At the population level, Barbieri et al. (2025) analyzed 3,918 acute myocardial infarction patients, integrating 4,686 AMI diagnoses and nearly half a million prescriptions into a large-scale knowledge graph comprising approximately 2,968 nodes and more than 800,000 edges. Their approach identified 249 potential drug–AMI associations, of which 63.4% corresponded to known adverse events. Jeong et al. (2025) extracted safety-related associations from 160,321 PubMed articles, identifying 111 candidate signals; 17 were validated in institutional EHRs, while nine represented previously undocumented findings. Finally, Zhao & Henriksson (2016) demonstrated that temporally weighted representations of clinical events consistently improved ADE prediction performance across datasets containing hundreds to thousands of patient visits.

### 3.4 Cross-study comparative analysis

Synthesis of findings revealed several dominant methodological trends across the literature:

- Dependence on structured EHR/claims data: All 15 studies used structured clinical data, with NLP components appearing only as auxiliary layers (Henriksson et al., 2016; Jeong et al., 2025; Zhao and Henriksson, 2016).
- Absence of pharmacovigilance datasets: No study used FAERS, VigiBase, or similar systems as analytic inputs, confirming an exclusive emphasis on real-world clinical data (Bagattini et al., 2019; Zhao et al., 2015).
- Frequent use of tree-based ensembles: Random Forests and XGBoost were among the most commonly applied methods (Chiu et al., 2024; Goyal et al., 2023; Zhao et al., 2015).
- Consistent role of regularized regression (LASSO): Applied in several studies for interpretability and feature selection (Li et al., 2014; Zhao et al., 2015).
- Limited use of deep learning: Typically applied only when temporal sequences or embedding-based representations were available (Bagattini et al., 2019; Morel et al., 2020; Zhao and Henriksson, 2016).
- Sparse adoption of external or temporal validation: Only a minority of studies implemented rigorous validation across cohorts or time periods (Lu et al., 2023; Zhao and Henriksson, 2016).
- Inconsistent application of explainability: Some studies used SHAP or KG-LIME (Patterson and Tatonetti, 2024), while many reported only predictive metrics.
- Minimal evaluation of fairness, calibration, or uncertainty: None of the studies conducted formal fairness assessments or uncertainty quantification.
- Limited evaluation of drug–drug interactions: Except for the mechanistic work of Datta et al. (2021) and the signal-validation pipeline of Jeong et al. (2025), few studies explored DDI-related ADE risks.

A further methodological aspect concerns class imbalance, which is intrinsic to adverse event prediction. Across the 15 included studies, explicit strategies for handling class imbalance (e.g., resampling techniques, synthetic minority oversampling, or cost-sensitive learning) were rarely described. For example, Zhao et al. (2015) and Henriksson et al. (2016) constructed balanced ADE datasets for model evaluation, thereby mitigating imbalance at the dataset level. In contrast, studies such as Chiu et al. (2024), Dil-Nahlieli et al. (2024), and Goyal et al. (2023) reported event frequencies but did not describe the application of specific imbalance-correction techniques. Overall, no consistent methodological framework for managing class imbalance was identified across the included literature.

Collectively, the results indicate that AI-driven ADE prediction research remains strongly anchored in real-world structured clinical data, with minimal integration of unstructured text or multimodal pharmacovigilance resources. Methodological characteristics and model performance metrics are presented in Table 2.

**TABLE 2:** Consolidated characteristics of the 15 studies included in the systematic review, including clinical domains, data sources, sample sizes, outcomes, machine-learning methods, performance metrics, validation strategies, and primary contributions.

Study (Year)	Clinical domain	Data source	Sample size	Outcome(s)	ML models	Best performance	Key numeric results	Validation	Key contribution
Chiu et al. (2024)	Nephrotoxicity (colistin)	Hospital EHR (ICU)	390 pts	KDIGO nephrotoxicity	LR, RF, XGBoost, SVM, MLP	AUROC 0.81	13.8% events	Train/test split; CV	ML-based prediction of colistin toxicity
Dil-Nahlieli et al. (2024)	Polypharmacy ADE risk	HMO EHR + claims	74,553 pts (28,412 polypharmacy)	ADE-related ED visits	Proprietary API ensemble vs. LR	AUROC 0.76	2,023 ADE ED visits; NRI ∼0.18	Dev/val cohorts	Data-driven ADE risk system for elderly
Zhao et al. (2015)	Multi-ADE detection	Hospital EHR	27 ADE datasets (hundreds episodes each)	ADEs (27 ICD-based)	LR, RF, SVM, etc.	Varies:RF > LR	AUROC per ADE dataset	Cross-validation	Structured EHR predictive framework
Henriksson et al. (2016)	ADE detection via NLP embeddings	EHR + clinical text	Massive corpus (700M tokens)	27 balanced ADE tasks	RF on multiple embedding spaces	Improved AUROC	9,046 ICD codes; 2.9M drug records	Cross-validation	Ensembles of distributed representations
Bagattini et al. (2019)	ADE prediction from sparse time-series	EHR time-series data	15 ADE datasets	Multiple ADE types	RF + temporal abstraction	Higher AUROC than baselines	Statistically significant improvements	Cross-dataset comparison	Sparse temporal feature modeling
Li et al. (2014)	Statin safety (MI risk)	EHR + claims	316,178 users	MI events	Cox models + HDPS	Stable HRs post-adjustment	10,745 MI events; 1.3M PY	Longitudinal follow-up	Controlling high-dimensional confounding
Datta et al. (2021)	NSAID liver injury + DDIs	National claims (France)	Simulations + large diabetic cohort	DILI	GBoost + TMLE	Higher power than SCCS	Simulations of 4,000 subjects × scenarios	Simulations + real data	ML to detect harmful DDI patterns
Morel et al. (2020)	AMI risk from drug exposures	National claims (France)	4,000 simulated + large diabetic cohort	AMI	ConvSCCS vs. SCCS	Lower bias	Better risk-window detection	Simulations + real data	Lagged ADE detection with ConvSCCS
Segal et al. (2019)	Medication error prevention	Hospital EHR (CDSS)	All hospital prescriptions	Prescription errors/potential ADEs	Probabilistic CDSS model	—	0.4% alerts; 85% valid; 43% changed orders	Prospective evaluation	ML-based prescribing safety alerts
Patterson and Tatonetti (2024)	MS treatment safety	EHR + knowledge graph	Cohort of MS patients	ADE risk profiles	Gradient boosting + KG-LIME	Comparable AUROC to baseline	Reduced explanation size; high fidelity	Internal validation	Knowledge-graph–constrained explanations
Lu et al. (2023)	Amiodarone thyroid dysfunction	Hospital EHR	2,544 pts	Thyroid dysfunction	LR, RF, XGBoost	AUROC 0.86	42.5% events	Train/val/test	Explainable ML for drug-induced dysfunction
Goyal et al. (2023)	SSRI-associated bleeding	All of Us EHR	11,408 pts	Major bleeding	LR, RF, XGBoost	C-stat 0.78	706 events (6.2%)	Internal validation	ML prediction of SSRI bleeding risk
Barbieri et al. (2025)	Drug safety in AMI	Italian EHR + claims	3,918 pts	AMI-related drug signals	Network ML	—	249 signals; 63.4% known AEs	Retrospective cohort	Network analysis for safety signal detection
Jeong et al. (2025)	Severe SADRs from PK-DDIs	NLP + multi-EHR	Literature: 160k articles; EHR validation	Severe ADRs	NLP extraction + LR validation	17 validated signals	111 extracted; 9 novel	Multi-database validation	PK-DDI signal detection pipeline
Zhao and Henriksson (2016)	Temporal modeling of ADE risk	EHR event sequences	Multiple ADE datasets	ADEs	Ensemble trees w/time weights	AUROC improved by weighting	Hundreds–thousands visits/dataset	Cross-dataset tests	Importance of temporal dynamics

Abbreviations: ADE, adverse drug event; ADR, adverse drug reaction; EHR, electronic health record; NLP, natural language processing; ML, machine learning; AUROC, area under the receiver operating characteristic curve; DDI, drug–drug interaction; SCCS, self-controlled case series; CDSS, clinical decision support system; SHAP, shapley additive explanations; NRI, net reclassification improvement; ICU, intensive care unit; KDIGO, Kidney Disease: Improving Global Outcomes; LR, logistic regression; RF, random forest; SVM, support vector machine; MLP, multilayer perception; HMO, health maintenance organization; ED, emergency department; ICD, international classification of diseases; NSAID, Non-Steroidal Anti-Inflammatory Drug; MI, myocardial infraction; PK-DDI, Pharmacokinetic Drug-Drug Interaction; SADR, Severe Adverse Drug Reaction; HDPS, High-Dimensional Propensity Score; DILI, Drug-Induced Liver Injury; PY, Person-Years.

## 4 Discussion

Across the 15 included studies, 12 (80%) relied exclusively on structured EHR or claims data, while 3 (20%) incorporated NLP-derived representations in addition to structured data (Henriksson et al., 2016; Jeong et al., 2025). Within the 12 structured-only studies, routinely collected variables were predominantly used, including laboratory values, coded diagnoses, medication exposure histories, and demographic indicators. Chiu et al. (2024) used structured ICU EHR data to predict colistin-induced nephrotoxicity, while Dil-Nahlieli et al. (2024) developed a risk-alert system for ADE-related emergency department visits using medication histories and comorbidity profiles from a large EHR-based cohort. Even studies employing more complex feature spaces, specifically Li et al. (2014) with high-dimensional confounder adjustment and Bagattini et al. (2019) with temporal abstractions, remained fundamentally grounded in structured EHR or claims data. Only 3 studies incorporated NLP components, including distributional embeddings trained on approximately 700 million clinical tokens (Henriksson et al., 2016) and literature mining of 160,321 PubMed abstracts with subsequent EHR-based validation of candidate signals (Jeong et al., 2025). None of the included studies used spontaneous reporting systems such as FAERS or VigiBase, and study implemented a multimodal framework combining EHR, NLP, and pharmacovigilance data.

No shared benchmarks datasets, outcomes, or validation protocols were used across the included studies which contrasined direct comparison between methods. Across studies, performance was reported using non-uniform metrics, including AUROC or C-statistics (e.g., Chiu et al., 2024; Dil-Nahlieli et al., 2024; Goyal et al., 2023; Lu et al., 2023), effect estimates such as hazard ratios following high-dimensional adjustment (Li et al., 2014), and bias or risk-window detection metrics in self-controlled simulation frameworks (Datta et al., 2021; Morel et al., 2020). Among studies reporting AUROC or C-statistics, best performance ranged from approximately 0.76 (Dil-Nahlieli et al., 2024) and 0.78 (Goyal et al., 2023) to approximately 0.81 (Chiu et al., 2024) and 0.86 (Lu et al., 2023). Assessment beyond purely internal validation was uncommon; cross-dataset or multi-database validation was reported in 3 of 15 studies (Bagattini et al., 2019; Jeong et al., 2025; Zhao and Henriksson, 2016). These discrepancies underscore the urgent need for unified evaluation frameworks in ADE prediction research. Beyond discrimination metrics such as AUROC or C-statistics, several important aspects of model evaluation were insufficiently addressed across the included studies. Calibration assessment, uncertainty quantification, and evaluation of clinically meaningful decision thresholds were rarely reported. While discrimination reflects ranking ability, inadequate calibration or poorly defined risk thresholds may substantially limit clinical applicability. The limited reporting of these complementary evaluation dimensions suggests that many ADE prediction models remain at a performance-assessment stage rather than readiness for real-world deployment.

Explainability was addressed inconsistently across the included studies. Lu et al. (2023) explicitly implemented SHAP-based feature attribution to provide clinically interpretable explanations of model predictions in amiodarone-induced thyroid dysfunction, while Patterson and Tatonetti (2024) introduced KG-LIME, a knowledge-graph–constrained explanation framework designed to reduce explanation complexity while preserving predictive fidelity. In contrast, the majority of studies, including Chiu et al. (2024), Dil-Nahlieli et al. (2024), Goyal et al. (2023), and Zhao et al. (2015), primarily reported performance metrics without detailed analysis of model interpretability mechanisms. Given the increasing regulatory and clinical emphasis on transparent and accountable AI systems in healthcare, the limited and uneven integration of explainability approaches across the reviewed literature may represent a barrier to broader clinical adoption. From a regulatory perspective, explainability is increasingly recognized as a prerequisite for high-risk AI systems in healthcare environments. Models intended for clinical decision support may require traceable logic, interpretable feature attribution, and transparent validation procedures to satisfy emerging governance and accountability frameworks. The uneven adoption of explainability approaches observed in the included studies may therefore limit not only clinical trust but also regulatory acceptability.

A further methodological observation concerns the limited incorporation of unstructured electronic health record data through natural language processing (NLP). Among the 15 included studies, only Henriksson et al. (2016), Zhao and Henriksson (2016), and Jeong et al. (2025) explicitly integrated text-derived representations alongside structured data. In the remaining studies, modeling relied exclusively on coded diagnoses, prescriptions, laboratory values, or administrative claims. Given that clinically relevant information such as symptom descriptions, narrative documentation of adverse events, medication changes, and contextual patient factors are often recorded in free-text clinical notes, this limited use of NLP represents a structural constraint in current ADE modeling approaches. More systematic integration of unstructured EHR data may enhance signal detection, improve phenotypic precision, and strengthen risk stratification models. Future research should therefore explore hybrid architectures combining structured clinical variables with robust text-mining pipelines to better capture the full informational richness of real-world clinical data.

Limitations of the study include the heterogeneity of reporting across the included articles, which restricted the feasibility of quantitative synthesis and prevented direct metric-level comparison. Additionally, reproducibility was constrained by incomplete reporting of feature engineering procedures, data preprocessing pipelines, and hyperparameter optimization strategies in several studies. Open code availability or detailed computational documentation was uncommon, limiting independent replication and external benchmarking. Across studies, implementation detail was variably reported, particularly for feature engineering and model selection procedures, which constrained reproducibility. Since meta-analysis was not feasible and the evidence base comprised 15 methodologically heterogeneous studies, conclusions should be interpreted with appropriate caution regarding generalizability. Notably, only 1 of 15 studies reported prospective evaluation in a live clinical decision support context (Segal et al., 2019).

An additional limitation concerns the search strategy. The literature search was restricted to a single biomedical database (PubMed/MEDLINE), which may have introduced selection bias and limited the comprehensiveness of the retrieved evidence. Although PubMed provides extensive coverage of biomedical research, relevant studies indexed exclusively in other databases such as Scopus or Web of Science may not have been captured. Consequently, some eligible studies might have been inadvertently omitted.

## 5 Conclusion

This systematic review highlights that AI-based adverse drug event prediction remains predominantly grounded in structured electronic health records, with limited integration of NLP methods and no use of pharmacovigilance databases. While ensemble and regularized models show consistent promise, external validation, explainability, and methodological transparency remain insufficient across studies. Strengthening these aspects will be essential for translating AI-driven drug safety tools into clinically reliable and generalizable applications.

## Data Availability

Publicly available datasets were analyzed in this study. No original datasets were generated in this study. Data were derived from previously published articles cited in the manuscript.
